# Determinants of the lethality of climate-related disasters in the Caribbean Community (CARICOM): a cross-country analysis

**DOI:** 10.1038/srep11972

**Published:** 2015-07-08

**Authors:** Aisha N. Andrewin, Jose M. Rodriguez-Llanes, Debarati Guha-Sapir

**Affiliations:** 1 Ministry of Health and Social Development, Government of Anguilla, West Indies; 2 Centre for Research on the Epidemiology of Disasters, Institute of Health and Society, Université catholique de Louvain, Brussels, Belgium

## Abstract

Floods and storms are climate-related hazards posing high mortality risk to Caribbean Community (CARICOM) nations. However risk factors for their lethality remain untested. We conducted an ecological study investigating risk factors for flood and storm lethality in CARICOM nations for the period 1980–2012. Lethality - deaths versus no deaths per disaster event- was the outcome. We examined biophysical and social vulnerability proxies and a decadal effect as predictors. We developed our regression model via multivariate analysis using a generalized logistic regression model with quasi-binomial distribution; removal of multi-collinear variables and backward elimination. Robustness was checked through subset analysis. We found significant positive associations between lethality, percentage of total land dedicated to agriculture (odds ratio [OR] 1.032; 95% CI: 1.013–1.053) and percentage urban population (OR 1.029, 95% CI 1.003–1.057). Deaths were more likely in the 2000–2012 period versus 1980–1989 (OR 3.708, 95% CI 1.615–8.737). Robustness checks revealed similar coefficients and directions of association. Population health in CARICOM nations is being increasingly impacted by climate-related disasters connected to increasing urbanization and land use patterns. Our findings support the evidence base for setting sustainable development goals (SDG).

In the last three decades (i.e., 1983–2013) 6,354 floods and storms were reported worldwide affecting approximately four billion people and resulting in almost 1.8 million injured and 647,812 dead^1^. However, knowledge about the risk factors associated with mortality due to these climate-related hazards is limited.

Demographic risk factors such as age, gender, ethnicity^2^,^3^,^4^ are perhaps the most consistently investigated, though results vary depending on context. A few studies have examined proximate risk factors at the individual level e.g. type of housing^5^,^6^ and seeking of shelter^6^; being in a motor vehicle at the time of the event^7^; living in coastal provinces^8^. Some studies have attempted to encompass more distal factors such as socioeconomic status^5^ and country-level characteristics such as income level and region^9^. While room exists for more epidemiological research on these risk factors, the paucity is greatest for the areas of highest mortality risk. Moreover, the need to formulate post-2015 Sustainable Development Goals (SDGs) which should be evidence-based^10^, increases the onus for studies to consider widely the distal societal factors which may be a consequence of current developmental policies. This is the case for the Caribbean, one of the world regions most frequently hit by tropical cyclones and flooding due to the accompanying storm surges^11^ and where the mortality risk associated with major climate-related hazards is considered to be amongst the highest^12^,^13^.

However, the risk factors for deaths during floods and storms in this region are unknown. Similarly, these risk factors are unknown for the member countries of the sub-regional entity known as the Caribbean Community (CARICOM). The CARICOM was established in 1973 to promote economic integration and cooperation and coordinate foreign policy among member states^14^. The fifteen current members are Antigua & Barbuda, Bahamas, Barbados, Belize, Dominica, Grenada, Guyana, Haiti, Jamaica, Monserrat, St. Kitts and Nevis, St. Lucia, St. Vincent and the Grenadines, Suriname, Trinidad and Tobago for an estimated population of over 16 million (Fig. 1). These countries also share a regional coordinating body for disaster response – the Caribbean Disaster Emergency Management Agency (CDEMA)^15^.

This paper aims to elucidate risk factors for mortality due to storms and floods i.e the lethality of these events in CARICOM countries.

## Results

### Decadal trends in climate-related disasters and study covariates

Between 1980 and 2012, 200 floods and storms were reported and 125 (63%) involved deaths. One hundred thirteen (57%) occurred between 2000 and 2012 and the proportion of lethal events was also higher for this period compared with 1980–1989 and 1990–1999. Storms numbered 129 (65%) and consistently accounted for the higher proportion of events across the decades. Events occurred in all countries except Monserrat; 75 (38%) occurred in Haiti and this was the highest proportion of all the countries. All other countries contributed each less than 14% of the total share. Except for the proportion of females, median country-level characteristics for the corresponding years of the events showed wide variation within and across the decades (Table 1).

### Determinants of lethal climate-related disasters

Of the explanatory variables initially considered, hospital beds and physicians per 1,000 population (as proxies for emergency medical systems) were dropped due to insufficient data. Flood and storm magnitudes had many missing values and were not considered for further analysis.

Twenty potential explanatory variables remained of which six variables were retained after bivariate analysis (Table 2) and multi-collinearity checks: % of the population 65 and older (p < 0.05); agricultural land (p < 0.001); land where elevation is below 5 meters (p < 0.05); urban population (p < 0.05); urban population growth (p < 0.01); period (p < 0.001) for 1980–1989 versus 2000–2012 (Table 2).

After backward elimination the final model consisted of four variables. Three were positively associated with lethality due to floods and storms: agricultural land (p < 0.01); % urban population (p < 0.05) and 2000–2012 compared with 1980–1989 (p < 0.05; Table 3). The fourth, % of land where elevation is below 5 meters, had a negative coefficient though the result was not significant at p < 0.05.

### Robustness checks

We applied our final model to a subset excluding Haiti in order to assess its potential influence on our findings. We also applied the model separately to floods and storms and to events occurring from 2000 to 2012. The directions of association were the same when the final regression model was applied to these subsets (Table 4). Additionally, the coefficients for agricultural land were positive and statistically significant (p < 0.05) for storms, islands and 2000–2012. It was also considerably smaller for the subset excluding Haiti although this was not statistically significant. The urban population coefficient was positive and statistically significant (p < 0.05) and considerably larger for 2000–2012. We obtained a similarly larger coefficient for floods but this result was not statistically significant. Coefficients for 2000–2012 (reference 1980–1989) were positive and statistically significant (p < 0.05) for islands and the subset excluding Haiti.

## Discussion

Our study alludes to land use (increases in land dedicated to agriculture) and urbanization (as increase in people living in urban areas) as two determinants of lethality to floods and storms in the CARICOM member countries, also adjusting for time as a decadal effect. Although they did not always reach statistical significance, our subset analyses confirmed our main findings, which were also coincident with the scant available literature on this topic^16^,^17^.

In multivariate regression analyses, Maqueo *et al.* analysed the relationship between the components of human, built, social, and natural capitals on mortality due to hurricanes worldwide. They found a negative association between mortality by hurricanes and the area covered by semi-altered ecosystems (p < 0.01) and GDP (p < 0.05)^16^. The authors concluded that a combination of infrastructure and relatively well preserved natural ecosystems (semi-altered ecosystems) might offer a good protection service against the impact of hurricanes in terms of preservation of human lives. In this study only one record per country was used. In a qualitative analysis of Central America and the Caribbean, Navarette *et al.* analysed the causal loops of three syndromes they identified to be associated with hydro-meteorological disasters in these regions. Ecosystem degradation was one of the syndromes and the authors pointed to Haiti (and El Salvador) as the most likely candidates to present a causal relation between deforestation and local flooding impacts^17^.

The positive association we found with lethal events and the percent of agricultural land, our proxy for land use, may be taken as consistent with the findings of those two studies. Our larger coefficient and positive and statistically significant findings for storms show further consistency with the Maqueo study. Furthermore, whereas the Maqueo study investigated hurricanes only, our findings indicate that this association holds true for floods as well and both climate-related disasters considered together for our CARICOM population. The notably smaller coefficient for the subset excluding Haiti is quantitative evidence which seems to concur with the findings of the Navarette study and provides an impetus for further research. Smaller samples in subset analyses however preclude definitive conclusions. On the other hand, the significant association with three of the subsets and the generally similar coefficient across subsets clearly suggest that these associations are valid.

The other two syndromes identified by the Navarette study pointed to the importance of “breaking urbanization cycles marked by the absence of effective land-use planning which lead to the occupation of hazardous areas by poor people”. Globally, urbanization continues to increase and unplanned urbanization increases exposure to natural hazards thereby increasing the increase risk of mortality and other adverse outcomes^18^. Urbanization, reflected as the proportion of the population living in urban areas, was high in this sample. Indeed, the Caribbean is one of the most highly urbanized regions in the world^19^. Our positive and statistically significant association between lethality and increasing urbanization was found for the full dataset as well as for the subset of events between 2000 and 2012. These findings are therefore consistent with the current paradigm on urbanization and natural hazards.

Our findings for 2000–2012 compared to the other decades are consistent with predictions of exacerbations, either in frequency or intensity, of climate-related hazards^20^,^21^,^22^. On one hand, more climate-related disaster events were reported in the most recent decade (even considering the two extra years in this decade) compared to 1980–1989 and 1990–1999 decades. On the other hand, the proportion of lethal climate-related disasters was statistically significant in 2000–2012 compared to 1980–1989. The impact of improved reporting with potential selection bias towards over-reporting of lethal events, however, also needs to be borne in mind when interpreting this result.

Our study has some limitations. First, our findings are not proof of causation but association. These findings were however robust in multivariate analysis, controlling for time (decade), and in subset analyses. Second, it is noteworthy to avoid making inferences at individual level as this analysis used grouped data at national level. Third, it is possible that the study’s power was inadequate to detect additional variables of significance to the study’s outcome due to the sample size. This is inherent to most ecological studies. At the minimum, our analyses, which draw from a very wide pool of potential factors, provide early insights not only into the most important determinants of lethality, but those with potentially far-reaching policy implications. Fourth, we did not analyse mortality rates as we lacked the data to surmise the part of the population at risk reliably across the countries. Instead we focused our study on analyzing climate-related disasters causing any death, as we consider disaster lethality to be a good indicator of public health impact. Finally, although we were confident in the use of the mortality data, as this is widely held to be the most reliable of the disaster outcomes data, these data do not reflect deaths due to long-term mortality which may be associated with the factors investigated here.

Our study alludes to the paucity of research on suitable proxies to measure the complex constructs associated with mortality risk in hydro-meteorological disasters and disasters in general. This speaks not only to the inherent construct validity of measures but also their applicability and consistency across various contexts. Although an important body of research exists on the specific attenuating and protective effect of natural coastal systems against tsunamis and storm surges^23^,^24^, scant empirical research has been dedicated to understand comprehensively the factors explaining mortality due to climate-related hazards using widely accepted frameworks^25^,^26^,^27^.

To our knowledge, this is the first study investigating quantitatively the regional determinants of lethality of disasters in the Caribbean. Current strategies for human development may produce collateral damage in terms of additional loss of life directly due to these climate-related disasters, and this effect remained in our analysis after controlling for a decadal effect. Certainly more research is needed to understand in-depth the aspects of current land use and urbanization increasing the likelihood of lethal disasters in this region, especially by means of micro-level studies.

Our findings can assist in the prioritization of action certainly, in the setting of the regional research agenda as relates to Disaster Risk Reduction and Mitigation. In a broader context, it is equally important to analyse these patterns in other areas of the world. This could enhance our understanding of the specific factors that are common and particular for certain regions, and strengthen the evidence base for effective solutions, especially in the context of ongoing development of SDGs^10^.

## Methods

### Review of mortality risk factors due to floods and storms in the Caribbean

We searched PubMed using the keywords “flood* Caribbean”, “storm* Caribbean”, “hurricanes Caribbean”, “hydrometereological Caribbean”, “tropical cyclone Caribbean”, as well as the following combination: (“mortality” OR “deaths”) AND (flood* OR storm* OR hurricane*) AND (“Caribbean” OR “CARICOM”). We conducted similar searches in Google Scholar. We found no studies that examine the risk factors for death during floods and/or storms in Caribbean countries. Our literature search also included six systematic reviews^2^,^3^,^4^,^9^,^28^,^29^. We found a general dearth of epidemiological studies investigating underlying risk factors for deaths during floods^2^,^3^,^4^,^9^ and storms^28^,^29^, especially distal societal factors. Moreover, none of the reviews included studies done on any of the countries in our CARICOM cohort. Finally, we contacted the Caribbean Health and Research Council of the Caribbean Public Health Agency and the Caribbean Disaster Management Agency and both entities confirmed the lack of data and epidemiological research on the topic and expressed this as an area where research is needed for the region.

### Study design and conceptual framework

We conducted an ecological study investigating the risk factors for lethality of floods and storms in the CARICOM countries from 1980 to 2012. For this purpose we rigorously selected proxy indicators for our risk factors based on existing and appropriate vulnerability frameworks. In Cutter’s Hazard of Place Model, vulnerability is considered both in terms of biophysical risk and the social response within a specified geographic domain that can be a geographic space where vulnerable people and places are located, or a social space that is one identifying people more vulnerable in those locations. The model also posits that vulnerability is not fixed but is instead changeable with time based on changes in risk, mitigation, and the contexts within which the hazards occur^25^. In later work, Cutter *et al.* used the following county-level socioeconomic and demographic indicators to construct an index of social vulnerability to environmental hazards: socioeconomic status, gender, race/ethnicity, age, development, employment loss, rural/urban, residential property, infrastructure and lifelines, renters, occupation, family structure, education, population growth, medical services, social dependence, and special needs^26^.

More recently Birkmann and other experts in vulnerability research methodologies developed the World Risk Index^27^. The index is comprised of a number of indicators grouped by four components: exposure to natural hazards; susceptibility as a function of public infrastructure, housing conditions, nutrition, and the general economic framework; coping capacities as a function of governance, disaster preparedness and early warning, medical services, social and economic security; adaptive capacities to future natural events and climate change.

Borrowing from those vulnerability constructs, we identified hazard characteristics and country-level indicators (for the given year of the event) as explanatory variables for the lethality of floods and storms in CARICOM countries. As per the Cutter model, we also investigated a decadal/temporal effect as an additional explanatory variable. The aim was not for inter-country comparison but to get insights into characteristics associated with lethality from a sub-regional perspective. Data availability ultimately determined the proxy indicators used (Table 5).

### Data sources

The sources used for the study were the Emergency Database, EM-DAT for the disaster-related data^1^ and World Bank (WB) database^30^ for country indicators for the corresponding years of the disaster events.

EM-DAT, maintained by the Centre for Research on the Epidemiology of Disasters (CRED), is one of the most recognized and widely-used international natural disasters databases available within the public domain^1^. Events due to the given hazards are classified as disasters and included in the database based on established criteria. The database captures the date, location and magnitude of disasters; categorizes them by sub-group, type and sub-types and provides information on certain impacts including the number of persons killed, injured and affected.

Flood and storms are two disaster types under the hydro-meteorological sub-group of natural disasters. Floods are sub-typed as general flood, flash flood, storm surge/coastal flood. Storms are sub-typed as tropical cyclones or local storms. EM-DAT captures magnitude as maximum wind speed in Km/hr and area affected in km2 for storms and floods respectively^1^. The number of persons killed comprises persons confirmed as dead and persons missing and presumed dead.

For the purpose of this study, we extracted data for floods and storms for CARICOM countries for the defined period of study. We included the number of persons killed as the outcome of interest, excluding persons injured and affected.

The World Bank maintains an open access global database on development indicators based on data obtained mainly from member countries and compiled using established methodologies^30^. We used the following country indicators for the corresponding years of the disaster events as proxies to measure our vulnerability constructs: the population percentages by sex and age group (0 to 14, 15 to 64, 65 and older) defined our demographic parameters. Population density, urban population and urban population growth percentages, and the percent of the land area where elevation is below 5 meters were proxies for population exposure. The percent of land used for agriculture was a proxy for land use; and the percent of the population with access to improved sanitation facilities was a proxy for infrastructure. GDP per capita and country income level (lower, lower middle, upper middle or high) were proxies for wealth. The number of telephone lines and mobile subscriptions per 100 people were proxies for information access for early warning. Infant and under-five mortality rates were proxies for level of development and hospital beds and physicians per 1,000 population were proxies for emergency medical systems.

We constructed our main outcome of interest, lethality, by dichotomizing the number of persons killed as zero killed versus any killed. We subdivided the period into three -1980 to 1989, 1990 to 1999, and 2000 to 2012 - as a proxy to investigate a decadal or temporal effect. We also added a category classifying countries as islands or coastal territories.

### Statistical methods

We first investigated bivariate associations between lethality and twenty explanatory variables (Table 2). We retained those with p values 0.05 or less for multivariate regression analysis using a generalized linear model (GLM) with a quasi-binomial distribution in order to model over-dispersion of the data^31^. High multi-collinearity among explanatory variables can be a major challenge for this study type. We used a conservative Variance Inflation Factor threshold (VIF) threshold of four to remove multi-collinear variables^32^. Some authors recommend that the interval for choosing a critical p-value which determines a stopping rule for stepwise regression should be 0.05 ≤ α ≤ 0.50^33^. We performed backward elimination based on p-values of 0.3^33^,^34^. Odds ratios (ORs) with their 95% confidence intervals (CI) were calculated^31^. All tests were two tailed and alpha level 0.05. The analyses were conducted using R software (Version 3.1.0)^35^.

## Additional Information

**How to cite this article**: Andrewin, A. N. *et al.* Determinants of the lethality of climate-related disasters in the Caribbean Community (CARICOM): a cross-country analysis. *Sci. Rep.* **5**, 11972; doi: 10.1038/srep11972 (2015).

## Figures and Tables

**Figure 1:**
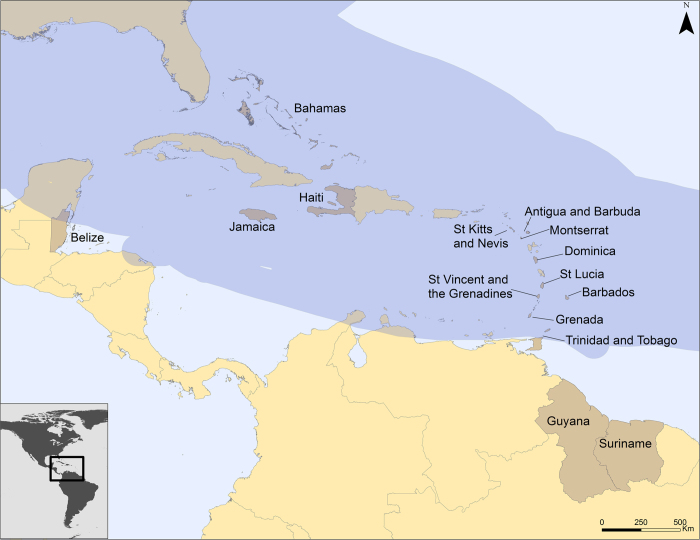
CARICOM countries within the Caribbean and regional belt of major hurricanes. CARICOM nations are highlighted in light brown; major hurricane belt emphasized in dark blue. The map was created in ArcGIS 10.2.2 software (ESRI Inc., Redlands, CA, USA) using data from the paths of tropical cyclones and major hurricanes from 1851 to 2004 (available at: http://www.mapcruzin.com/natural-disaster-shapefiles/hurricane-arcgis-shapefile-download.htm). Hurricane belt estimated using a 100-km buffer.

**Table 1:** Frequency and distribution of variables by decade.

Variable	1980–1989 (n = 43)	1990–1999 (n = 44)	2000–2012 (n = 113)	Total (n = 200)
Lethality of disaster				
Any deaths, No. (%)	18 (42%)	22 (50%)	85 (75%)	125 (62.5%)
Disaster type				
Storm, No. (%)	22 (51%)	32 (73%)	75 (66%)	129 (64.5%)
Flood, No. (%)	21 (49%)	12 (27%)	38 (35%)	71 (35.5%)
Country				
Haiti, No. (%)	14 (33%)	9 (20%)	52 (46%)	75 (37.5%)
Other, No. (%)	29 (67%)	35 (80%)	61 (54%)	125 (62.5)
Country type				
Island, No. (%)	42 (98%)	40 (91%)	99 (88%)	181 (91%)
Coastal, No. (%)	1 (2%)	4 (9%)	14 (12%)	19 (9%)
Population density, median (interquartile range)	218.9 (195.2–247.5)	226.3 (129.3–276.7)	290.8 (195.0–340.7)	247.1 (162.6–321.5)
Missing	0	0	7	7
Population				
Percentage population 0–14 years, median (interquartile range)	40.3 (36.8–42.9)	34.4 (31.9–41.3)	35.8 (29.4–37.7)	36.3 (30.4–38.9)
Percentage population 15–64 years, median (interquartile range)	53.5 (53.1–56.2)	58.6 (53.8–62.3)	59.8 (58.1–63.5)	59.1 (56.1–62.1)
Percentage population 65 years and older, median (interquartile range)	5.1 (4.0–6.9)	5.9 (4.1–7.3)	4.5 (4.2–7.2)	4.5 (4.2–7.2)
Percentage population female, median (interquartile range)	50.7 (50.7–50.9)	50.6 (50.3–50.9)	50.6 (50.5–50.7)	50.6 (50.6–50.8)
Missing (age group & sex)	6	7	3	16
Percentage of total land dedicated to agriculture, median (interquartile range)	44.2 (32.8–58.0)	29.5 (15.8–46.2)	43.4 (18.0–60.1)	43.1 (20.5–60.6)
Missing	0	0	7	7
Percentage of total land area where elevation is below 5 meters, median (interquartile range)	8.0 (3.9–15.7)	9.4 (7.1–21.8)	3.9 (3.9–9.5)	7.1 (3.9–15.7)
Percentage urban population, median (interquartile range)	32.9 (26.4–39.7)	34.2 (32.5–48.1)	47.4 (43.9–52.0)	44.9 (33.2–52.0)
Percentage annual urban population growth, median (interquartile range)	2.4 (0.7–6.1)	1.5 (0.7–3.0)	2.4 (0.8–4.7)	2.2 (0.7–4.5)
Percentage of population with access to improved sanitation facilities, median (interquartile range)	NA	80.4 (64.4–87.5)	70.3 (25.1–82.9)	79.6 (25.1–86.3)
Missing	43	0	11	54
Telephone lines (100 people), median (interquartile range)	3.6 (0.6–6.6)	16.5 (8.2–29.3)	10.4 (1.1–20.6)	9.6 (1.1–20.8)
Missing	1	0	0	1
Mobile phone subscriptions (100 people), median (interquartile range)	0.0 (0.0–0.0)	0.3 (0.0–1.4)	41.9 (16.0–69.2)	5.4 (0.0–47.4)
Missing	1	2	0	3
GDP per capita, median (interquartile range)	1398.4	3563.0	3495.3	3298.9
	(1179.3–2152.9)	(2005.6–6398.9)	(627.7–5250.6)	(732.2–5395.2)
Missing	14	1	0	15
Country income level				
Low, No. (%)	14 (33%)	9 (20%)	52 (46%)	75 (37.5%)
Lower middle, No. (%)	1 (2%)	1 (2%)	3 (3%)	5 (2.5%)
Upper middle, No. (%)	20 (47)	16 (36%)	41 (36%)	77 (38.5%)
High, No. (%)	8 (19%)	18 (41%)	17 (15%)	43 (21.5%)
Infant MR (per 1,000 live births), median (interquartile range)	27.4 (23.9–104.9)	20.8 (15.5–30.6)	22.6 (17.1–64.4)	24.4 (17.4–64.4)
Missing	2	0	0	2
Under5 MR (per 1,000 live births), median (interquartile range)	33.4 (29.5–152.3)	23.1 (18.8–34.9)	25.0 (19.5–88.1)	29.0 (20.1–88.1)
Missing	2	0	0	2

NA = not applicable. MR = mortality rate.

**Table 2:** Bivariate associations of flood and storm disaster lethality with each selected variable.

Variable		Coefficient	OR	95% CI	*p*
Population density (per km^2^)		0.002	1.002	0.999–1.004	0.158
Population 0–14 (%)		0.031	1.032	0.979–1.089	0.241
% of population 15–64		−0.023	0.978	0.915–1.044	0.500
% of population 65 and older		−0.177	0.837	0.705–0.991	0.041
% of population female		0.065	1.067	0.628–1.854	0.812
Improved sanitation facilities (% with access)		−0.029	0.971	0.956–0.985	<0.0001
Infant mortality rate		0.014	1.014	1.003–1.025	0.014
Under-5 mortality rate (per 1,000 live births)		0.010	1.010	1.003–1.017	0.008
Telephone lines (per 100 people)		−0.028	0.972	0.952–0.992	0.008
Mobile phone subscriptions (per 100 people)		0.003	1.003	0.995–1.010	0.521
Agricultural land (%)		0.029	1.029	1.015–1.044	<0.0001
Land area where elevation is below 5 meters (% of total land area)		−0.018	0.982	0.966–0.998	0.028
Urban population (%)		0.019	1.020	1.001–1.040	0.047
Urban population growth (% annual)		0.221	1.247	1.090–1.438	0.002
GDP per capita (constant 2005 US$)		−0.0001	1.000	1.000–1.000	0.045
Disaster type					
Flood	*Reference*				
Storm	0.035		1.036	0.565–1.883	0.909
Disaster subtype					
Flash flood	*Reference*				
General flood	0.811		2.250	0.260–15.081	0.413
Local storm	13.468		7.061	0.000–NA	0.988
Storm surge	−1.099		0.333	0.001–11.767	0.507
Tropical cyclone	−0.618		0.539	0.075–2.496	0.467
Country type					
Coastal	*Reference*				
Island	0.685		1.983	0.758–5.260	0.162
Period					
1980–1989	*Reference*				
1990–1999	0.329		1.389	0.593–3.286	0.451
2000–2012	1.439		4.216	2.016–9.032	<0.0001
Income level					
High	*Reference*				
Low	1.800		6.052	2.641–14.506	<0.0001
Lower middle	−1.058		0.347	0.016–2.646	0.368
Upper middle	0.669		1.953	0.915–4.244	0.088

NA = not available; OR = odds ratio.

**Table 3:** Multivariate model of flood and storm disaster lethality with relevant determinants.

Variable	Coefficient	OR	95% CI	*p*
Agricultural land (% of land area)	0.031	1.032	1.013–1.053	0.002
Land area where elevation is below 5 meters (% of total land area)	−0.015	0.985	0.96–1.010	0.247
Urban population (% of total)	0.028	1.029	1.003–1.057	0.033
Period				
1980–1989	*Reference*			
1990–1999	0.694	2.002	0.811–5.040	0.137
2000–2012	1.311	3.708	1.615–8.737	0.003

†VIF < 4 and after backward elimination. VIF = variance inflation factor. OR = odds ratio.

**Table 4:** Robustness checks by comparison of multivariate logistic regression models on study subsets.

Variable	All data (n = 193)	Haiti excluded (n = 123)	Storms (n = 125)	Floods (n = 68)	Island (n = 174)	2000–2012 (n = 106)
Agricultural land (% of land area)	0.031**	0.019	0.036*^b^	0.031	0.033*^c^	0.029*^e^
Land area where elevation is below 5 meters (% of total land area)	−0.015	−0.016	−0.007	−0.088	−0.012	−0.033
Urban population (% of total)	0.028*	0.027	0.020	0.051	0.024	0.051*^f^
Period						
1980–1980	*Reference*	*Reference*	*Reference*	*Reference*	*Reference*	NA
1990–1999	0.849	0.836	0.132	1.434	0.733	NA
2000–2012	1.310**	1.089*^a^	0.997	1.213	1.295*^d^	NA

*p < 0.05. **p < 0.01; ^a^OR, 95% CI (2.969, 1.052–8.818); ^b^OR, 95% CI (1.037, 1.010–1.067); ^c^OR, 95% CI (1.034, 1.004–1.065); ^d^OR, 95% CI (3.651, 1.562–8.754); ^e^OR, 95% CI (1.029, 1.003–1.056); ^f^OR,95% CI (1.052, 1.009–1.104).

**Table 5:** Summary of vulnerability concepts, explanatory variables and data sources.

Vulnerability concepts^a^	Proxy indicators	Source
Biophysical vulnerability		
Event characteristics	Disaster type – storm versus flood	EM-DAT
	Disaster sub-type^b^	EM-DAT
Exposure of population	Land area where elevation is below 5 meters (% of total land area)^c^	WB
	Urban population (% of total)	WB
	Urban population growth (annual %)	WB
	Population density (per km^2^)	WB
Geographical characteristics		
Country type	Island versus coastal country	Constructed
Land use	Agricultural land (% of land area)	WB
Social vulnerability		
Population characteristics	Population 0–14 (% of total)	WB
	Population 15–64 (% of total)	WB
	Population 65 and above (% of total)	WB
	Population, female (% of total)	WB
Infrastructure	Improved sanitation facilities (% of population with access)	WB
Information access	Telephone lines (per 100 people)	WB
	Mobile cellular subscriptions (per 100 people)	WB
Poverty	GDP per capita (current US$)	WB
	Country income level (low, lower middle, upper middle, high)	WB
Level of development	Mortality rate, infant (per 1,000 live births)	WB
	Mortality rate, under 5 (per 1,000 live births)	WB
Time		
Time period	1980–1989, 1990–1999, 2000–2012	Constructed

^a^Inspired by Cutter’s Hazard of Place Model^25^,^26^ and Birkman *et al*’s World Risk Index^27^.

^b^In EM-DAT storms are sub-classified as either tropical cyclones or local storms and floods sub-classified as general flood, flash flood, storm surge/coastal flood.

^c^Used in lieu of population living in areas where elevation is below 5 meters (% of total population).
